# Modeling Consequences of COVID-19 and Assessing Its Epidemiological Parameters: A System Dynamics Approach

**DOI:** 10.3390/healthcare11020260

**Published:** 2023-01-13

**Authors:** Ateekh Ur Rehman, Syed Hammad Mian, Yusuf Siraj Usmani, Mustufa Haider Abidi, Muneer Khan Mohammed

**Affiliations:** 1 Department of Industrial Engineering, College of Engineering, King Saud University, Riyadh 11421, Saudi Arabia; 2 Advanced Manufacturing Institute, King Saud University, Riyadh 11421, Saudi Arabia

**Keywords:** COVID-19, SEIR model, pandemic, mathematical modeling, virus

## Abstract

In 2020, coronavirus (COVID-19) was declared a global pandemic and it remains prevalent today. A necessity to model the transmission of the virus has emerged as a result of COVID-19’s exceedingly contagious characteristics and its rapid propagation throughout the world. Assessing the incidence of infection could enable policymakers to identify measures to halt the pandemic and gauge the required capacity of healthcare centers. Therefore, modeling the susceptibility, exposure, infection, and recovery in relation to the COVID-19 pandemic is crucial for the adoption of interventions by regulatory authorities. Fundamental factors, such as the infection rate, mortality rate, and recovery rate, must be considered in order to accurately represent the behavior of the pandemic using mathematical models. The difficulty in creating a mathematical model is in identifying the real model variables. Parameters might vary significantly across models, which can result in variations in the simulation results because projections primarily rely on a particular dataset. The purpose of this work was to establish a susceptible–exposed–infected–recovered (SEIR) model describing the propagation of the COVID-19 outbreak throughout the Kingdom of Saudi Arabia (KSA). The goal of this study was to derive the essential COVID-19 epidemiological factors from actual data. System dynamics modeling and design of experiment approaches were used to determine the most appropriate combination of epidemiological parameters and the influence of COVID-19. This study investigates how epidemiological variables such as seasonal amplitude, social awareness impact, and waning time can be adapted to correctly estimate COVID-19 scenarios such as the number of infected persons on a daily basis in KSA. This model can also be utilized to ascertain how stress (or hospital capacity) affects the percentage of hospitalizations and the number of deaths. Additionally, the results of this study can be used to establish policies or strategies for monitoring or restricting COVID-19 in Saudi Arabia.

## 1. Introduction

Severe acute respiratory syndrome coronavirus 2 (SARS-CoV-2) is a novel virus associated with the emerging coronavirus disease 2019 (COVID-19), which spread quickly throughout the world and caused a global pandemic [1]. The first pandemic of this century began in late December 2019 in Wuhan, China, where it was initially discovered. COVID-19 has since spread worldwide since the beginning of 2020; as of 29 November 2022, the World Health Organization (WHO) coronavirus dashboard reported more than 640 million positive cases globally [2]. When compared to earlier coronavirus epidemics of SARS-CoV (severe acute respiratory syndrome coronavirus) and MERS-CoV (Middle East respiratory syndrome coronavirus), COVID-19 exhibits distinctive epidemiological characteristics. The fact that COVID-19 is extremely contagious and that its precise nature is still unclear are among the factors contributing to the global pandemic. Numerous transmission events took place via direct human-to-human contact among people who had no or minor symptoms, including in nosocomial and community settings [3]. To curb the spread of the virus, most nations enacted lockdown procedures and stringent social confinement [4]. From a clinical perspective, these methods are a great way to prevent virus spread, but they have a negative impact on the economy and society. Therefore, under any situation, a complete lockdown for an extended period of time is not desirable to maintain the economic standing of a nation. These lockdowns have disturbed the global supply chain and distribution networks of goods and products. Therefore, the aftereffects of such lockdowns can be considered dangerous [5]. Therefore, these two distinct aspects of governmental policies, i.e., total lockdown and societal health, should be balanced appropriately. This is only possible when the pertinent information is available to decision-makers at an appropriate time. Moreover, from the point of view of healthcare strategy, understanding the pattern of a disease’s transmission and being able to predict it over time are crucial elements because they can lessen the social and economic effects, as well as prevent deaths.

Researchers, practitioners, and decision-makers are very much interested in developing a variety of models to comprehend the trajectory of the pandemic and to devise efficient control tactics [6]. In the literature, a variety of models have been employed, including mathematical models [7,8,9,10,11,12,13], statistical models [14,15,16,17,18], network-based models [19,20,21], artificial intelligence (AI) models [8,22,23,24], and simulation models [25,26,27]. The output of these models has exhibited extreme importance for decision-makers in controlling the pandemic’s spread and its adverse effects [28]. These models can compare several scenarios depending on the available data in order to forecast the path of the pandemic, as well as propose measures for managing it. Several such models have been reported in the literature, with some of the widely known ones being the susceptible–infected–recovered model [29,30,31], curve-fitting model [32], extended–susceptible–infected–recovered model [33], susceptible–exposed–infected–quarantined–dead–hospitalized–recovered model [27], susceptible–unascertained–cases–pre-symptomatic infectiousness–exposed–infectious–recovered model [34], susceptible–infected–diagnosed–ailing–recognized–threatened–healed–extinct model [35], and susceptible–exposed–asymptomatic–infected–hospitalized–recovered–dead due to COVID-19 infection–susceptible model [36]. Although these are all mathematical models, their complexity increases and applicability decreases as the amount of data increases, necessitating an exponential growth in computational power. Similarly, large volumes of data are required for training AI models [37]. Consequently, simulation-based models have also emerged as effective solutions [38].

Hence, in this research, with the help of a susceptible–exposed–infected–recovered epidemic (SEIR) model, a simulation-based model is developed to comprehend the dynamics of COVID-19 in Saudi Arabia. This study examines how epidemiological variables such as seasonal amplitude, societal awareness influence, and waning time can be modified in order to accurately estimate COVID-19 scenarios, such as the number of infected persons on a daily basis in Saudi Arabia. The optimal state of epidemiological variables and the impact of COVID-19 are determined using system dynamics (SD) modeling and design of experiment (DOE) techniques. The aim is to match the simulation model with empirical data to determine its suitability for assessing the efficacy of the Saudi government’s control measures, as well as forecast the disease’s future dynamics in Saudi Arabia on the basis of various scenarios. The proposed model takes into account the dynamic nature of person-to-person interaction behaviors, as well as the differences in susceptibility and infectiousness across persons. The results of this study can be useful for decision- makers to curb the spread and effects of the pandemic through proper planning. The presented study mainly contributes to the literature through the use of SD modeling and DOE approaches to determine the most appropriate combination of epidemiological parameters and the influence of COVID-19. Similarly, this study can help decision-makers to predict and correctly estimate the number of infected persons on a daily basis in the Kingdom of Saudi Arabia (KSA). The objective of this model is to ascertain how stress (or hospital capacity) affects the percentage of hospitalizations and the number of deaths. Additionally, several scenarios are investigated to predict the future dynamics of COVID-19 in KSA. Hence, this model can be used to comprehend the current state of COVID-19, as well as monitor and control its impact. The main contribution of this study is its application of an enhanced SEIR model in the form of a susceptible–exposed–infected–hospitalized–isolated–recovered–susceptible (SEIHIsRS) framework to realistically simulate the pandemic’s spread. The model established in this work considers both isolated and vaccinated persons, in contrast to past studies that were mostly based on the classic SEIR model so as to simplify the simulation. This study attempts to recreate the state of COVID-19 as precisely as possible by considering a number of aspects that were disregarded in previous research. Specifically, this work takes into account factors such as the influence of lockdowns, social awareness, seasonality, and vaccine efficacy, along with standard criteria such as latent time, recovery time, and mortality rate. Prior studies did not simultaneously study all of these variables. The integration of DOE with Vensim modeling, particularly for COVID-19, is an efficient method to calibrate the simulation model in accordance with the real scenario; however, this approach was not previously applied in the literature.

This study is structured into five main sections. The research background and study objectives are covered in Section 1. Section 2 provides a review of the literature to identify research gaps that assisted in defining the objectives. The methodology comprising a description of the model and experiments is presented in Section 3. Lastly, the results and conclusions are summarized in Section 4 and Section 5, respectively.

## 2. Literature Survey

There has been a significant volume of research in the literature on the dynamics and progression of contagious diseases such as COVID-19. For example, Mwalili et al. [39] adopted the fourth and fifth-order Runge-Kutta method to depict only the COVID-19 transmission dynamics and does not describe the disease severity and deaths. He et al. [40] studied the impact of quarantined and hospitalization in predicting the dynamics of COVID-19. The model was applied to the actual COVID-19 data of Hubei province. In their model, the rate of infectious to hospitalized individuals and the recovered rate of quarantined infected individuals were estimated using a particle swarm optimization algorithm with actual data of recovered and hospitalized. It was found that with seasonality and stochastic infection, the system could generate chaos. The dynamics of the system were found to be different for a different set of parameters. Whereas, Annas et al., 2020 [41] considered vaccination and isolation factors as model parameters. Their results showed that the vaccination could enhance disease healing and maximum isolation could slow down the spread of COVID-19 in Indonesia. Yarsky [42] incorporated a genetic algorithm to fit the population-dependent parameters to forecast the spread of COVID-19 for different states in the US. The most important parameter that could vary from state to state was considered to be the contact rate. Other parameters such as transmission probability, death rate, diagnostic test eligibility, and the test result period were found to vary to a lesser extent between the states. The use of a genetic algorithm was found to result in good agreement with the model and actual data. Chen et al. [43] proposed a model to establish the relationship between the spread of COVID-19 and mitigation measures to control it. The data was segmented into eight different periods with corresponding SEUIR models for each period and estimated the transmission rate and reduced rate for each period. The performance of the proposed model was tested for the US COVID-19 data obtained from the world meter. Similarly, Zhang et al. [44] proposed a model to study the effect of intra-city, and inter-city population movements as well as medical investments on the spread of COVID-19 in three cities of Hubei Province, China. Reproduction numbers of the proposed model were derived theoretically using the next-generation matrix method and the effect of selected parameters on the spread of COVID-19 was simulated. Liu et al. [45] studied the effectiveness of the control measure adopted during COVID-19 using area-based exposure to infections during travel and quarantine. The proposed model considered infections during travel and the effect of control measures such as social distancing, working from home, circuit breaker, and phase advisory on infection risk. The developed model was applied to assess Singapore’s COVID-19 response concerning the national policies and transport policy. The movement of commuters between the zones was modeled using Singapore’s mass rapid transit data. After model calibration and parameter estimation, a long-term investigation of the COVID-19 pandemic along with a disease transmission dynamics model was presented.

Kamrujjaman et al. [46] developed a COVID-19 epidemic model using first-wave data from Italy and Spain. The fit of the proposed model with real data was found to be good when tested using the least square method and residuals. Sensitivity analysis revealed the most sensitive model parameters as disease transmission rate, panic, tension/anxiety of susceptible and infected, natural death rate, and disease-induced death rate. Tello et al. [47] proposed a mechanism to monitor the dynamics of an epidemic in a prescribed region with a varying population using time-variant parameters of diffusion and transmission along with the data from health authorities regarding positive tests and deaths. Kiarie et al. [48] proposed a model to forecast the spread of the COVID-19 pandemic in Kenya. Their model parameters were estimated using historical data and model fit was evaluated. Yin et al. [49] proposed a population-based model to study the COVID-19 transmission dynamics in India during the first wave. The model was constructed considering the infection complexities, symptoms, and transmission pathways to perform a retrospective analysis of government policies such as lockdown, individual protection actions, testing, and screening. The model was calibrated using the reported data on daily infected, death, and recovered cases from various states of India. The analysis showed that the strict practice of individual protection methods is essential to moderate lockdown policy and mitigate the propagation of disease. Hatami et al. [50] considered the spatial heterogeneity of the pandemic and proposed a model with spatial extension to simulate and predict the dynamics of the COVID-19 delta variant in the Metropolitan Statistical Area in the US. The model was fitted with the daily data of COVID-19 cases and deaths from John Hopkins University. Thus, multiple models were developed considering various external covariates and relevant datasets such as mobility, pharmaceutical, and non-pharmaceutical interventions, demographics, and weather data to improve the robustness and predictive performance of the model. Phan et al. [51] developed a quantitative framework to estimate COVID-19 prevalence and predict virus transmission using wastewater-based surveillance data from the second wave pandemic data of three counties in Massachusetts. They presented a dynamic model that connects the viral load in wastewater with the total number of infected cases in the sewer shed. Sun et al. [52] focused on an asymptomatic/pre-symptomatic population and a symptomatic population to study the dynamics of COVID-19 in Japan. Furthermore, Carcione et al., 2020 [53] first implemented an SEIR model to determine the infected, recovered, and dead individuals in the Italian region of Lombardy. The model was calibrated with the data from Lombardy available online till 5 May 2020 which was then used to predict the dynamics of the epidemic. The analysis also showed the importance of isolation, social distancing, and knowledge of diffusion conditions to better understand the dynamics of the epidemic and stop the spread of disease. Feng et al., 2021 [54] incorporated an SEIR model to study the dynamics of the epidemic in Wuhan. Furthermore, neural networks-based artificial intelligence models were used to analyze the epidemic trend in non-Wuhan areas. The model was calibrated using the data from January to March 2020 obtained from the literature. The proposed SEIR and AI models effectively predicted the epidemic peaks and sizes in Wuhan and non-Wuhan areas respectively. The study also found that the control measures taken by the Chinese government helped in reducing the scale of the epidemic. Prem et al., 2020 [55] evaluated the effect of control strategies such as social distancing measures on the spread of the COVID-19 epidemic in Wuhan using SEIR modeling. The results showed that the control measures for social mixing in the population are effective in reducing the spread and delaying the peak of the epidemic. Chung and Chew, 2021 [56] studied the COVID-19 outbreak in Singapore using the SEIR model with multiplex and temporal networks. The study considered the complex human interactions such as social interactions in households and at the workplace in addition to the interactions between crowds, and social gatherings. The simulation results showed that the residents in densely populated areas were more susceptible and easily infected. The spread of infections in these areas could be uncontrollable without proper control measured.

It is evident from the literature that there are several variables whose values have been thoroughly investigated and documented in the literature. There are also some epidemiological variables, such as seasonal amplitude, social awareness impact, waning time, etc., which have not been studied in the past but can play a crucial role in understanding the dynamics of COVID-19. Therefore, these variables have been examined in this study using DOE and SD modeling so that the developed model could be changed to account for the COVID-19 trend in KSA. Furthermore, the values of already investigated variables, such as infection period, isolation time, recovery time, etc., are obtained from the literature or online sources. Additionally, initial trials or exploratory experiments, and expert opinions are used to obtain the values for some variables that are not available in the literature. For example, the initial hospitalization percentage, days to seasonal change, days to achieve social awareness, etc., are among these variables.

## 3. Model Description and Experiments

**Figure 1:**
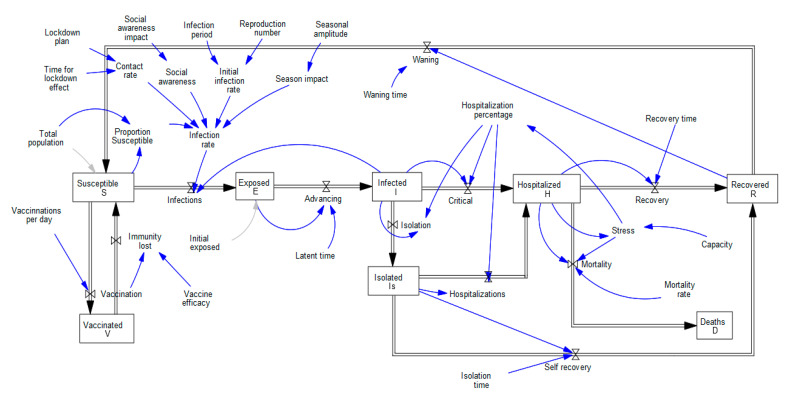
Stock and Flow diagram for the COVID-19 pandemic model.

In this research, modeling is undertaken using the SD approach [57], a simulation method for comprehending the nonlinear behavior of complicated systems that are frequently used for feedback loop assessment. The main elements of this modeling approach are stocks (represented by a box), flows (symbolized by valves with arrows), auxiliary, and delay components. A variation of the well-known compartmental disease diffusion model, known as the SEIHI_s_RS model and analogous to the models outlined in [58,59], is used to simulate a realistic pandemic spread situation. Figure 1 shows a high-level stock-flow schematic of the intended framework. The entire population is divided into five sections in this model, including Susceptible (S), Exposed (E), Infected (I), Hospitalized (H), and Isolated (I_s_)—Recovered (R). This model assumes that each compartment’s population composition is uniform. Additionally, no birth and natural death rates are considered, and there is no infection rate due to hospitalized patients.

The following discusses how the SEIHI_s_RS pandemic model works. Anyone who becomes infected (I) exposes the susceptible (S) people to the disease. The migration of the susceptible population to exposure (or the number of infections) relies on the infection rate. According to Equation (1), the infection rate (IR) is the result of the initial infection rate (IR_i_), contact rate (λ), proportion susceptibility (S_p_), seasonal impact (φ), and social awareness impact (α).

IR = IRi × λ × Sp × φ × α(1)

The infection rate in the absence of any additional influencing elements, such as social awareness, season, lockdown, etc., is known as the initial infection rate. It only takes into account the reproduction number (R_0_) i.e., the number of times the certain virus reproduces, and the infection period (IP), as indicated in Equation (2). The average number of infected contacts per infected person is known as the reproduction number, whereas the infection period refers to the duration of an individual’s infectiousness. The R_0_ in the present case is assumed to be 3.3 [60,61,62,63,64] while the IP is considered as 7 [65,66].

(2) $${\mathrm{IR}}_{i}=\frac{R_{0}}{\mathrm{IP}}$$

**Figure 2:**
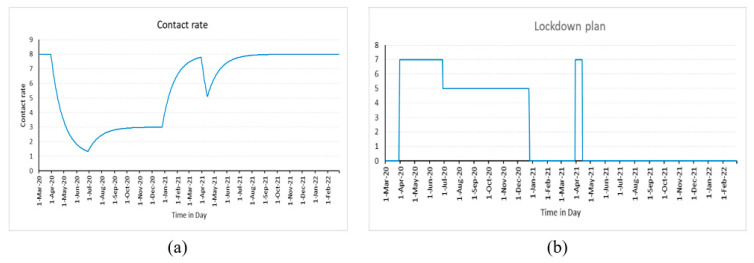
Behavior of (**a**) Contact rate; (**b**) Lockdown plan.

In the present case, it is presumable that the lockdown plan (β) to start and stop, as well as its time of impact (t_impact_), will affect the contact rate. It means that the contact rate is higher when there is no lockdown (it is supposed to be eight in the current experiment [67]), but it drops when there is a lockdown. The contact rate (λ) is represented by Equation (3) and its behavior which is represented by the exponential delay of the first order can be seen in the following Figure 2a. The duration of time after which the effects of lockdown are felt is known as the time of impact. The t_impact_ is obtained through experimentation.

λ = (λ_NL_ − DELAY1(β, t_impact_)) (3)

Figure 2b depicts the behavior of the variable β as defined by Equation (4). It means there will not be a lockdown for 30 days, at which point its value is seven. Consequently, after 120 days, the value of the β is reduced to five, and the cycle then repeats as predicted by Equation (4).

β = Step(7,30) − Step(2,120) − Step(5,300) + Step(7,395) − Step(7,410)(4)

The “proportion susceptibility” reflects the fraction of the initial population other than the susceptible. It depicts the people who are located in the less dense region of the social network. This component loses relevance and its value in cases when the entire population is presumed to be susceptible. The susceptible population in the present work is the entire population, hence the S_p_ is set at 1.

φ = 1 + ρ × (SMOOTH (PULSE TRAIN (T_s_, T_D_, T_R_, T_F_), d_season_)) (5)

**Figure 3:**
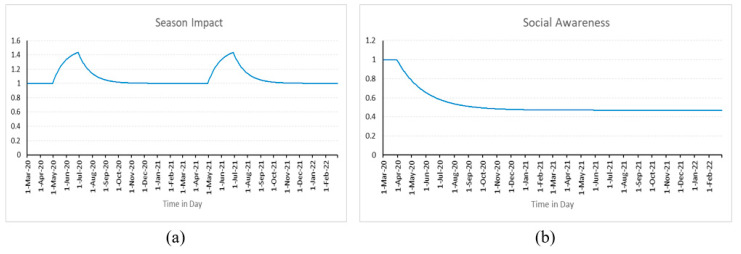
Behavior of (**a**) Seasonal impact; (**b**) Social awareness.

The inclusion of the seasonal impact considers the influence of the season on the progression of the pandemic. It is defined using the SMOOTH and PULSE functions as depicted in Equation (5). The season impact, according to the equation, begins on the 60th day and lasts for 60 days. This process repeats every 365 days, and since this model is created over two years, it ends after 21 months. The seasonal impact on the infection rate is considered using the SMOOTH function, which incorporates an exponential delay of the first order (Figure 3a). The variable “Days to seasonal change (d_season_)” is used to lessen the abrupt change and ease the impact of the seasons. After several preliminary experiments, the d_season_ is assumed to be 30 days in the current study. Furthermore, the variable ρ represents the seasonal amplitude and it is estimated through experimentation.

Social awareness is used to consider how the infection rate is impacted by actions like social distancing, mask use, frequent hand sanitization, etc. It implies that the spread of COVID-19 can be limited if the public is aware of its responsibilities. The model incorporates the effect of social awareness using Equation (6). Following the implementation of social awareness measures, it states that the impact will be 1 for 30 days before dropping to τ. The value of τ is estimated through experimentation and it represents the social awareness impact. The influence of social awareness on the infection factor is smoothed using the function DELAY1, as seen in Figure 3b. After the initial simulation runs, the variable d_achieve_ is fixed at 60 days, and it shows that the impact of social awareness on the infection rate steadily decreases to half in the 60 days.

α = 1 − DELAY1(Step (τ, 30), d_achieve_) (6)

The exposed individuals begin to experience symptoms after the latent time (L_T_) and move to the infected (I) compartment to seek healthcare. The patient is promptly moved to the Hospitalized (H) compartment if the hospital permit based on the availability of beds, medications, PPE kits, staff, etc.; otherwise, they proceed to the Isolation (I_s_) compartment and are permitted to continue in self-recovery. The admission rate or percentage of the Infected population that is hospitalized (HP) has been estimated. HP has been calculated in the developed model using the percentage of initial hospitalization (IHP) and the stress (σ). After the expert’s advice, the value of IHP is assumed to be 0.2 in the current model. The value of 0.2 indicates that 20 out of every 100 infected people are hospitalized. The percentage of infected individuals who are hospitalized, however, dramatically declines as the stress, which is defined as the ratio of the hospitalized to the capacity of the hospital (or healthcare facilities), increases. The burden or distress placed on the healthcare system as a result of overloading is conceptually referred to as stress. Thus, if the stress level is less than 0, the HP remains at the normal 0.2, but as soon as it rises above 0, it has a significant impact on the HP. This is done to ensure that patients only enter hospitals if they can receive the necessary care; otherwise, they move to isolation to recover on their own.

People in hospitals frequently recuperate following “Recovery time (R_T_)” and transition to the Recovered class (R). After an isolation time (I_ST_) longer than the R_T_, a percentage of the isolated infected patients achieve self-recovery. A certain number of isolated people are also hospitalized if their health deteriorates to a serious level. Some of the hospitalized patients also pass away depending on their stress level and mortality rate (μ). Those who have recovered lose their immunity to the disease and are once more vulnerable to it (probably as a result of disease strain mutation) after a considerable amount of time or the “Waning time (W_T_)”. This model also considers how immunizations affect the number of infected populations and it also assumes that vaccination immunity is lost depending on the vaccine efficacy (ζ). The model also considers the fact that immunization in KSA starts in mid of December 2020 [68]. The governing equations of this model for different compartments can be presented using Equations (7)–(13). The notations used in the model, their description, and their units are presented in Table 1.

(7) $$S=\underset{\mathrm{Immunity}\;\mathrm{lost}}{\underbrace{\left\{\left(1-\zeta \right)\times \left(\eta \right)\right\}}}+\underset{\mathrm{Waning}}{\underbrace{\frac{R}{W_{T}}}}-\underset{\mathrm{Infections}}{\underbrace{\left\{\left(I\right)\times \left(\mathrm{IR}\right)\right\}}}-\underset{\mathrm{Vaccination}\;\mathrm{per}\;\mathrm{day}}{\underbrace{\eta }}$$

(8) $$E=\left\{\left(I\right)\times \left(\mathrm{IR}\right)\right\}\;-\;\underset{\mathrm{Advancing}}{\underbrace{\frac{E}{L_{T}}}}$$

(9) $$E=\left\{\left(I\right)\times \left(\mathrm{IR}\right)\right\}\;-\underset{\mathrm{Advancing}}{\underbrace{\frac{E}{L_{T}}}}$$

(10) $$H=\;\left\{\left(I\right)\times \left(\mathrm{HP}\right)\right\}\;+\underset{\mathrm{Hospitalizations}}{\underbrace{I\times \mathrm{HP}\times 0.5}}-\underset{\mathrm{Mortality}}{\underbrace{\left\{H\times \mu \times \sigma \right\}}}-\underset{\mathrm{Recovery}}{\underbrace{\frac{H}{R_{T}}}}$$

(11) $$R=\frac{H}{R_{T}}+\underset{\mathrm{Self}\;\mathrm{recovery}}{\underbrace{\frac{I}{I_{\mathrm{ST}}}}}-\frac{R}{W_{T}}$$

(12) V=ηζ

(13) $$I_{s}=\{\left(I\right)\times \left(1-\mathrm{HP}\right)\}-\{I\;\times \;\mathrm{HP}\;\times \;0.5\}-\frac{I}{I_{\mathrm{ST}}}$$

### 3.1. Experimentation

The SD model is created in the commercial software Vensim^®^ (Ventana Systems, Inc., Harvard, MA, USA), and it is simulated using the Euler integration method with a time-step of 1. Vensim is the simulation program that predominantly provides continuous simulation for SD by offering a graphical modeling interface for stock-and-flow and causal loop diagrams where text-based equations can be included. There are several variables whose values have been thoroughly investigated and documented in the literature. Therefore, values for these variables, such as infection period, isolation time, recovery time, etc., are obtained from the literature or online sources. Initial trials or exploratory experiments and expert opinions are used to obtain the values for some variables that are not available in the literature. The initial hospitalization percentage, days to seasonal change, days to achieve social awareness, etc., are among these variables. Finally, some variables have not been studied in the past but can play a crucial role in understanding the dynamics of COVID-19. These variables are adjusted in this study using DOE so that the existing model could be changed to account for the national COVID-19 trend. As a result, this model can be utilized to comprehend the actual COVID-19 scenario in KSA as well as monitor and control the impact of COVID-19. The appropriate range for these parameters is chosen through preliminary runs. Table 2 shows the model tuning parameters and their levels, whereas Table 3 shows the remaining parameters with known or initially determined values.

The developed model is calibrated from the actual data by calculating the percentage difference in the total number of cases over 21 months. Around 243 simulation experiments are carried out in the calibration (or tuning). The model is run for each parameter combination for 21 months since the actual data for KSA is taken into consideration from March 2020 to November 2021. The COVID-19 scenario in KSA can be realized perfectly using this model (with idealized parameters) when the percentage difference is minimized at a specific set of parameters. Consequently, this model could be used to develop strategies and policies for reducing the COVID-19 spread and to research the impact of various policies on the COVID-19 spread. The values of parameters that have not been considered in DOE are presented in Table 3.

### 3.2. Impact of Policies Using the Established Model

Once the model has been adjusted for the KSA context, it is utilized to assess the impact of various policies on the number of hospitalizations per day and fatalities. The different policies that are investigated are as follows.

#### 3.2.1. Effect of Lockdowns

A lockdown is a state of confinement that compels individuals, a community, or an entire nation to remain in their current location. It restricts mobility or operations in a society while letting only those organizations operate regularly that provide essential goods and services. The extent of enforcement required in the implementation of lockdown can vary depending on necessity [73]. A lengthy lockdown may have highly negative effects on the economy. Additionally, there are also possibilities for long-term psychological repercussions including dissatisfaction, monotony, and worries about becoming sick, running out of supplies, etc. Therefore, the lockdown could have a detrimental effect rather than a good one depending on how well it is designed or executed. The impact of lockdown has been explored in this work so that proper lockdown policies can be developed at the appropriate moment.

#### 3.2.2. Impact of Social Awareness

Infectious transmission can be inhibited by social awareness and personal actions. These campaigns can take a variety of forms, from governmental rulings to societal pressure [74]. In the case of COVID-19, the results of public awareness campaigns and personal initiatives include better cleanliness habits (sanitization), the use of masks and personal protective equipment, social distancing, etc. These efforts demonstrate a population’s readiness to take part in infection prevention, which can help reduce obstacles to the execution of preventive policies. Social awareness can stop the disease from spreading, but it needs to be properly monitored and applied [75]. Therefore, to create effective policies for reducing COVID-19, the effect of social awareness has been researched in this study.

#### 3.2.3. Influence of Vaccination Efficacy

Throughout the COVID-19 period, many vaccinations have been developed, however, their efficacies vary significantly. For instance, Pfizer has a 95% efficacy rate, compared to 76% for Astra Zeneca [76]. This implies that the type of vaccine may also have a significant role in limiting the transmission of COVID-19. Therefore, the impact of various vaccine efficacies on infections, hospitalizations, and fatalities has been investigated in this work. This research enables us to comprehend how the effectiveness of immunizations affects the transmission of COVID-19.

## 4. Results and Discussions

**Figure 4:**
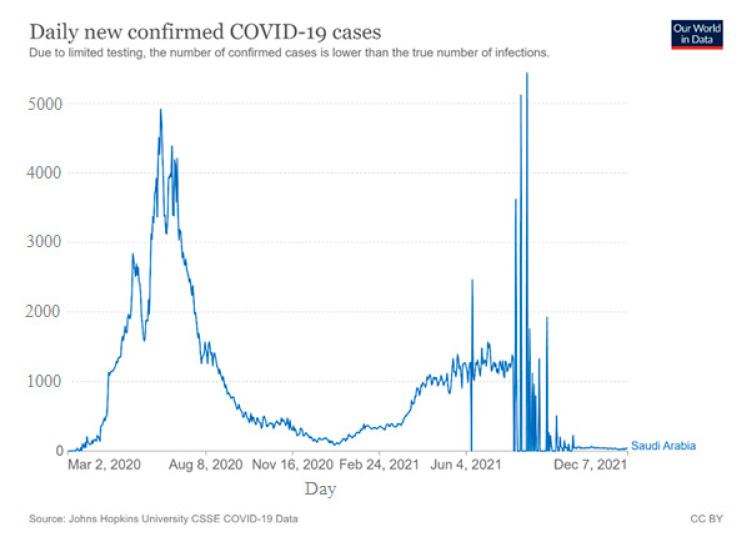
Actual Daily COVID-19 cases from March 2020 to November 2021 in KSA [72].

This study aims to identify appropriate parameter values (refer to Table 2) that reasonably fit the actual COVID-19 daily infection data. Therefore, the 21-month data for daily verified COVID-19 infected cases have been considered in this investigation. This information is obtained from open-access published data [72]. The historical confirmed infected cases from March 2020 to November 2021 are represented by the graph in Figure 4 below.

In Minitab software (Minitab 17, State College, PA, USA), a general full factorial DOE comprising 243 experiments is generated. The developed model is then put through a simulation to comprehend the dynamics of infected individuals for various parameter combinations. Then, for each simulation run, the daily infected cases are plotted and compared to the aforementioned historical actual data of daily infected cases. The error for each run is estimated and compared on average to identify the percentage difference. The lowest values and the best fitting parameter values for the three best and worst runs are presented in the following Table 4.

**Figure 5:**
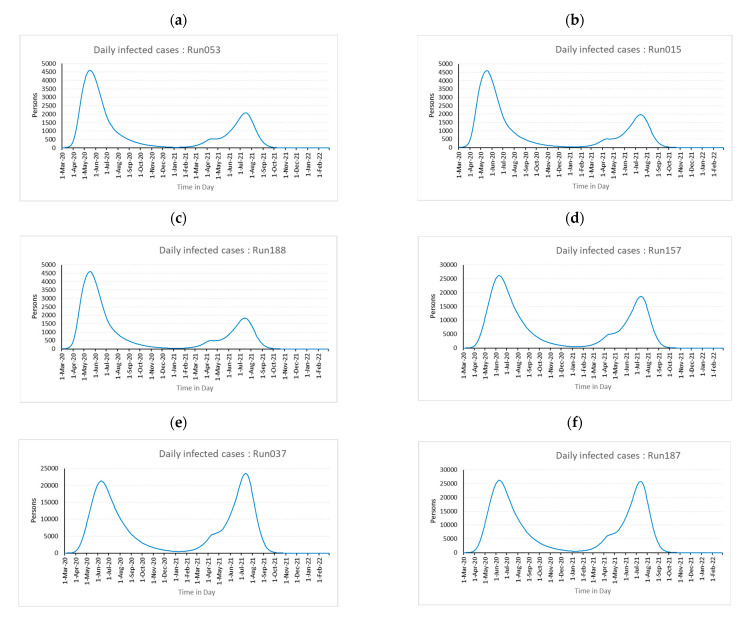
Daily infected cases for simulation (**a**) Run053, (**b**) Run015, (**c**) Run188, (**d**) Run037, (**e**) Run157, and (**f**) Run187.

The daily infected cases for each above simulation runs are plotted and are presented in Figure 5a–f.

From the above comparison of the model’s daily infected cases vs actual daily infected cases, it is observed that the model parameters set for Run053 are the best-fitted parameters to understand the impact of pandemic management policies. These parameters are 30 days (t_impact_), 75 persons (E initial value), 90 days (W_T_), high (τ), and significant (ρ). This model can be utilized to explain the dynamics of COVID-19 with the least percentage difference, once these parameter values are included together with additional parameter values gathered from literature and preliminary experiments.

There is a decline in the number of daily infected individuals after a few days of enforcing the lockdown. The effect of lockdown is observed to be 30 days in the KSA based on the DOE analysis of the established pandemic model mentioned above, and the initial value of those exposed should be considered 75 people. Similarly, to control and reduce the number of daily infected people, initiatives are taken by government agencies via educating and enforcing the public to maintain social distancing, making the use of masks and sanitizer compulsory in public places, etc. All of these initiatives are incorporated into τ, and three distinct levels of τ are used to test the model. For the example of KSA, the appropriate value of τ in this model is found to be 0.53. It is also apparent from the available literature and scientific facts that the season affects how contagious the pandemic virus is. This parameter is also considered as ρ in the model. Following the DOE study, the value of ρ is determined to be 0.5. Once the model is fine-tuned, it is used to study the effect of different policies on hospitalization and fatalities. Similarly, the influence of the efficacy of different vaccines and their effect on hospitalization and fatalities are also studied and discussed here below.

**Figure 6:**
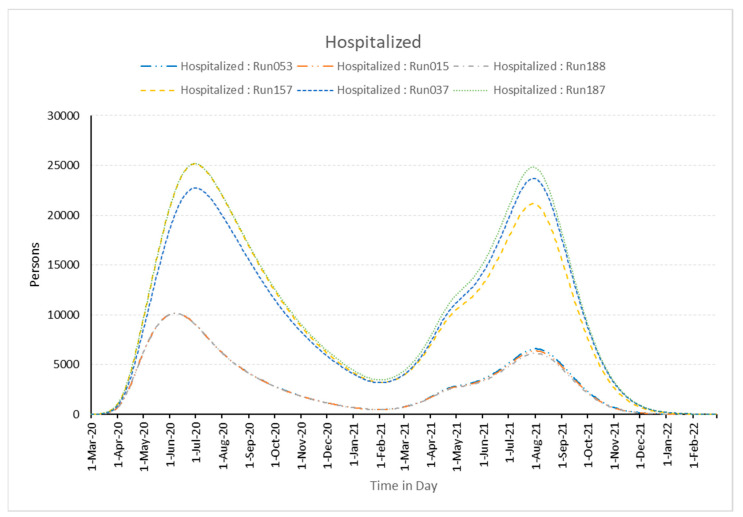
Number of persons hospitalized in simulation runs best-fitted runs (Run053, Run015, and Run188) and worst-fitted runs (Run037, Run157, and Run187).

**Figure 7:**
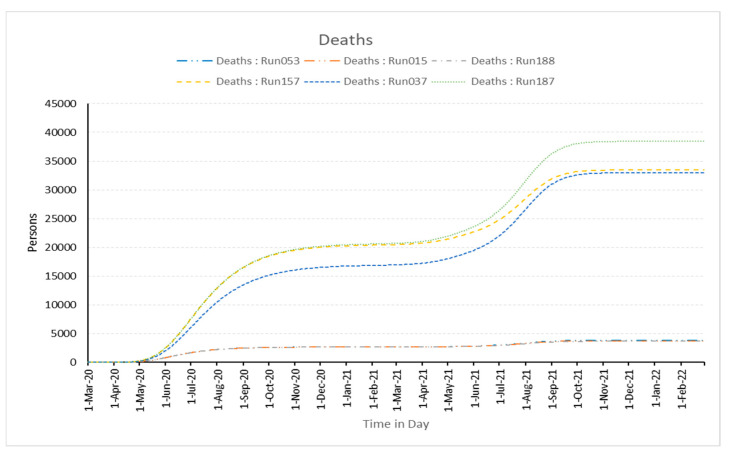
Number of deaths in simulation runs best-fitted runs (Run053, Run015, and Run188) and worst-fitted runs (Run037, Run157, and Run187).

The burden on hospitals during pandemics is primarily affected by government isolation measures and the daily infected population, which indirectly influences stress and the number of fatalities. Additionally, the chosen best and worst experiments and the related graphs shown below in Figure 6 and Figure 7 make this quite clear. This shows that the model is sensitive to changes in the parameters.

Decision-makers in government organizations must evaluate pandemic policies before choosing and putting them into practice. The various strategies are thus examined after choosing the parameters that best match the actual daily infected numbers for the KSA. The model with run 053 is chosen to examine the efficacy of vaccinations, societal awareness campaigns, and lockdown implementation procedures. The policies must be balanced. For example, a full lockdown will greatly affect the economy of the country. Aggressive social awareness measures will distress individuals and affect their mental health. Higher vaccine efficacy comes with higher vaccination costs. Therefore, it is important to analyze the different scenarios and understand their effect on the evolution of the pandemic.

**Figure 8:**
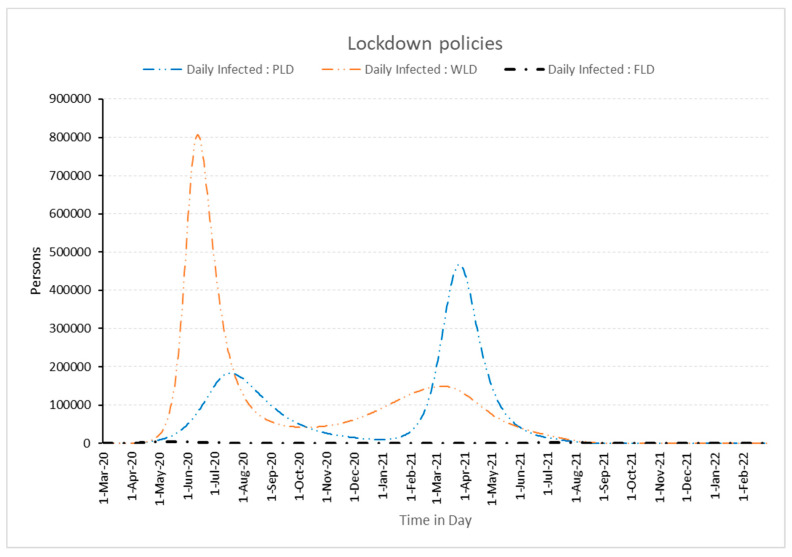
Impact of different lockdown policies on daily infected people.

From Figure 8, it can be observed that the number of cases without lockdown (WLD) increased in multiples of one hundred, while in partial lockdown (PLD), initially the number of the daily infected cases are downplayed, but subsequently, in the second seasonal wave impact it rises to double that of first wave infections. Similarly, when one follows a full lockdown policy, the number of daily infected cases is at a very low level in the thousands only. This supports the full lockdown policy adopted by WHO operational planning guidelines to support and control the pandemic effect.

**Figure 9:**
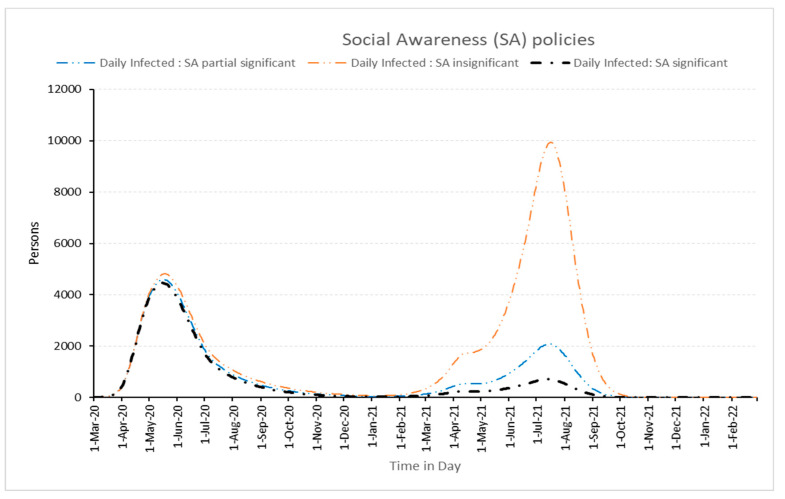
Impact of different social awareness efforts policies on daily infected people.

The success or failure of controlling any outbreak is not only depending upon lockdown measures but also requires public awareness in tackling pandemics. The majority of social awareness comes from media and preventive measures of pandemic awareness about diseases. As shown in Figure 9, social awareness is effective at the end of the pandemic cycle. This is obvious because making social awareness as described above takes a long time to reach a susceptible population. It is a cumulative effect and thus shows relative impact. One can observe that when social awareness is insignificant, the rise in infected persons will be in the very high range in the second wave. Thus, social awareness plays a significant role in controlling the pandemic.

**Figure 10:**
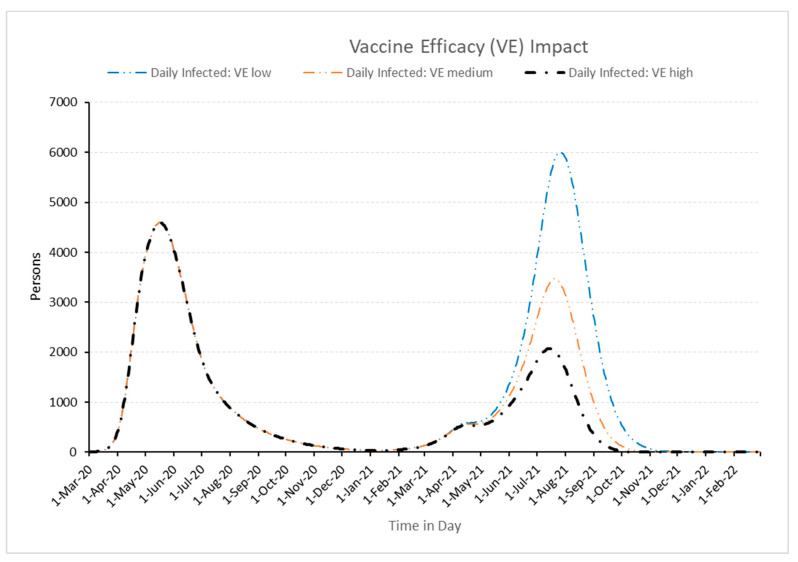
Impact of vaccine efficacy on daily infected people.

All vaccines approved by WHO for use have been through randomized clinical trials to test their quality, safety, and efficacy. To be approved, vaccines are required to have a high efficacy rate of 50% or above. A vaccine’s efficacy is measured in a controlled clinical trial and is a measure of how much the vaccine lowered the risk of becoming sick. It is evident that when a vaccine has high efficacy, it lowers the risk of becoming sick. Vaccines were not available in the earlier beginning stage of the pandemic, they were only developed and made available eight months after the pandemic started, which is seen in Figure 10. In earlier months of the pandemic, vaccine efficacy has no impact on the number of infected cases as the vaccines were in the development stage. The impact of vaccine efficacy on the infected case is seen in the later stages of the pandemic. It is worth noting that as vaccine efficacy increases from low to high, the number of infected people drops significantly. Thus, the policy to select effective vaccines by decision-makers has very high importance.

## 5. Conclusions and Future Work

It is essential to model the dynamics of the contagious COVID-19 virus to prevent its spread across the world. As a result, it is an effort in that direction. The objective of this work is to build a Susceptible-Exposed-Infected-Hospitalized-Isolated-Recovered-Susceptible model that can demonstrate how the COVID-19 outbreak spread throughout the KSA. It seeks to understand the critical COVID-19 epidemiological factors by using actual data. It employs SD modeling and the DOE to identify the most suitable combination of epidemiological variables and the impact of COVID-19. Some epidemiological factors, such as seasonal amplitude, social awareness impact, waning time, etc., which have not previously been investigated but can be vital to know the dynamics of COVID-19, should be taken into consideration. Consequently, the primary focus of this work has been on these variables. It is discovered from the relation of the model’s daily infected cases vs actual daily infected cases that the best-fit input variables to realize the implications of pandemic management policies are 30 days for t_impact_, 75 persons for E initial value, 90 days for W_T_, high social awareness impact, and greater value of seasonal amplitude. Thus, the proposed model can be effectively utilized to explain the dynamics of COVID-19 with a minimum percentage difference when the input values are appropriate.

It has been discovered that the number of daily affected people is decreasing only after a few days of imposing the lockdown. It means that the impact of the lockdown is seen 30 days after it is put into place in the KSA. This implies that the lockdown’s timing is vital for realizing its impact at the appropriate time before the situation goes out of control. The suitable value for the influence on social awareness is also found to be high. It shows that the steps taken by the government organizations, such as social distancing, the wearing of masks, frequent hand washing, etc., have a big impact on containing and reducing the spread of COVID-19 in KSA. It is also apparent that the pandemic virus’s contagiousness is significantly influenced by the season. It emphasizes the importance of additional precautions and stringent regulations when the virus may be more active and extra contagious.

It has also been observed that the full lockdown policy, along with an abiding and aware population (greater social awareness), considerably reduces any pandemic effect, particularly COVID-19. In addition, it is crucial to use vaccines that are effective enough to inhibit and stop the transmission of any contagious virus such as COVID-19. As a result, not just any vaccine should be selected, but a vaccine that can give the desired level of long-lasting immunity should be chosen. Additionally, it is critical for decision-makers in government entities to assess a variety of pandemic strategies before selecting and implementing them. The policies must be balanced because a complete lockdown is excellent, yet it can negatively impact the nation’s economy. Although aggressive social awareness campaigns are essential, at the same time they can irritate people and ruin their mental health. Similarly, more expensive vaccinations come with increased vaccine efficacy. Henceforth, it is crucial to evaluate the various scenarios and understand how they will affect the dynamics of the pandemic. The developed model will be made more relatable in future research by incorporating natural birth and death rates. The model will be further refined by taking into account infection rates associated with hospitalization, age-dependent transmission rates, and virus mutation or variants. Incorporating a strong healthcare inventory management model makes the SEIR model more useful by ensuring that the necessary healthcare requirements are available when they are required. Thus, the inventory supply chain model will be integrated with the SEIHIsRS model to evaluate various inventory supply scenarios and simulate the demand for desirable medical items such as pharmaceuticals, vaccinations, beds, etc. at the appropriate moment.

## Figures and Tables

**Table 1:** Notations deployed in the SEIHI_s_RS model.

Symbol	Name	Description	Units
S	Susceptible	People who have not yet been exposed to the infection but are likely to be vulnerable to the virus	Person
E	Exposed	Persons who have been exposed to the virus but have not yet begun to infect others	Person
I	Infected	Individuals who are infectious and spread the disease	Person
H	Hospitalized	People treated with infectious diseases in the hospital	Person
I_s_	Isolated	Persons who are not admitted to a hospital for treatment but are instead kept in disconnection	Person
R	Recovered	Patients recuperated from COVID-19	Person
D	Deaths	Individuals who have succumbed to COVID-19 infection	Person
V	Vaccinated	Individuals who have been immunized for COVID-19 infection	Person
	Capacity	The number of individuals that can be effectively treated by the healthcare system.	Person
IR_i_	Initial Infection Rate	The infection rate in the absence of any additional influencing elements	1/Day
IR	Infection Rate	The rate at which infection takes place and it depends on several influencing factors	1/Day
HP	Hospitalization Percentage	Admission rate or percentage of the infected population that is hospitalized	1/Day
IHP	Initial Hospitalization Percentage	A normal proportion of infected patients admitted to hospitals	1/Day
μ	Mortality rate	Number of deaths among COVID-19 patients each day	1/Day
IP	Infection Period	Duration of an individual’s infectiousness	Day
L_T_	Latent time	Duration after which the COVID-19 exposed individual becomes contagious	Day
t_impact_	Time of impact	Duration after which the effects of lockdown are felt	Day
d_season_	Days to seasonal change	The timeframe during which the seasonal impact takes place	Day
d_achieve_	Days to achieve	The timeframe during which the effect of social awareness takes place	Day
T_s_	Start time	Day of the year when season impact begins	Day
T_D_	Season impact duration	The period during which the effects of the season persist	Day
T_R_	Season impact repeat	Day of the year when season effect recurs	Day
T_F_	Final time	The final time of the simulation	Day
I_ST_	Isolation time	Duration of patients in seclusion after which self-recovery takes place	Day
R_T_	Recovery time	Duration of patients in hospital after which recovery takes place through treatment	Day
W_T_	Waning time	Duration after which recovered people lose their immunity and are once more vulnerable to COVID-19	Day
η	Vaccination per day	Daily doses of vaccines administered	Person/Day
λ	Contact rate	The rate at which individuals come in contact with each other, resulting in disease spread	Dimensionless
λ_NL_	No lockdown contact rate	Contact rate when there is no lockdown	Dimensionless
S_P_	Proportion susceptibility	Fraction of the initial population other than susceptible who are located in the less dense region of the social network	Dimensionless
φ	Seasonal impact	Seasonal effects on the spread of the pandemic	Dimensionless
ρ	Seasonal amplitude	The extent to which the seasons influence the disease’s spread	Dimensionless
α	Social awareness	Effect of measures such as social distancing, usage of masks, frequent hand sanitization, etc.	Dimensionless
τ	Social awareness impact	The extent to which social awareness influences the control of disease’s spread	Dimensionless
R_0_	Reproduction number	Number of times the virus reproduces	Dimensionless
β	Lockdown plan	Establishing the lockdown plan with start and end times	Dimensionless
σ	Stress	The burden on the healthcare system as a result of overloading	Dimensionless
ζ	Vaccine efficacy	Effectiveness of immunization	Dimensionless

**Table 2:** Variables and their corresponding levels chosen for refining the model.

Parameter	Levels		
E (Initial)	75 persons	100	12
ρ	Insignificant	Partial significant	Significant
τ	Low	high	Very high
t_impact_	15 days	30	45
W_T_	90 days	180	365

**Table 3:** Parameter settings for experimentation.

Symbol	Values	Symbol	Values
S	34,810,000	L_T_	14 [69]
I	0	d_season_	30
H	0	d_achieve_	60
I_s_	0	I_s_T	15 [70]
R	0	R_T_	20 [71]
R_0_	3.3 [60,61,62,63,64]	μ	0.003 [71]
D	0	S_p_	1
IHP	0.2	ζ	0.95
IP	7 [65,66]	η	Data [72]

**Table 4:** Three best and worst runs parameters.

Parameter↓ Runs→	Best Three Runs in the Best Fitting Order			Worst Three Runs in the Best Fitting Order		
	053	15	188	037	157	187
t_impact_	30	30	45	45	45	15
E (Initial value)	75	75	100	125	125	75
W_T_	90	180	90	180	90	90
τ	high	high	low	low	low	low
ρ	significant	significant	significant	significant	significant	partial significant

## Data Availability

Not applicable.
